# *Piriformospora indica* Stimulates Root Metabolism of *Arabidopsis thaliana*

**DOI:** 10.3390/ijms17071091

**Published:** 2016-07-08

**Authors:** Nadine Strehmel, Susann Mönchgesang, Siska Herklotz, Sylvia Krüger, Jörg Ziegler, Dierk Scheel

**Affiliations:** 1 Department of Stress and Developmental Biology, Leibniz Institute of Plant Biochemistry, Weinberg 3, 06120 Halle (Saale), Germany; susann.moenchgesang@ipb-halle.de (S.M.); siska.herklotz@pflanzenphys.uni-halle.de (S.H.); sylvia.krueger@ipb-halle.de (S.K.); 2 Department of Molecular Signal Processing, Leibniz Institute of Plant Biochemistry, Weinberg 3, 06120 Halle (Saale), Germany; joerg.ziegler@ipb-halle.de

**Keywords:** plant, fungus, interaction, exudates, roots, leaves, metabolite profiling, liquid chromatography/mass spectrometry (LC/MS), gas chromatography/mass spectrometry (GC/MS)

## Abstract

*Piriformospora indica* is a root-colonizing fungus, which interacts with a variety of plants including *Arabidopsis thaliana*. This interaction has been considered as mutualistic leading to growth promotion of the host. So far, only indolic glucosinolates and phytohormones have been identified as key players. In a comprehensive non-targeted metabolite profiling study, we analyzed *Arabidopsis thaliana*’s roots, root exudates, and leaves of inoculated and non-inoculated plants by ultra performance liquid chromatography/electrospray ionization quadrupole-time-of-flight mass spectrometry (UPLC/(ESI)-QTOFMS) and gas chromatography/electron ionization quadrupole mass spectrometry (GC/EI-QMS), and identified further biomarkers. Among them, the concentration of nucleosides, dipeptides, oligolignols, and glucosinolate degradation products was affected in the exudates. In the root profiles, nearly all metabolite levels increased upon co-cultivation, like carbohydrates, organic acids, amino acids, glucosinolates, oligolignols, and flavonoids. In the leaf profiles, we detected by far less significant changes. We only observed an increased concentration of organic acids, carbohydrates, ascorbate, glucosinolates and hydroxycinnamic acids, and a decreased concentration of nitrogen-rich amino acids in inoculated plants. These findings contribute to the understanding of symbiotic interactions between plant roots and fungi of the order of Sebacinales and are a valid source for follow-up mechanistic studies, because these symbioses are particular and clearly different from interactions of roots with mycorrhizal fungi or dark septate endophytes

## 1. Introduction

*Piriformospora indica* is a root-interacting endophytic fungus and has been found in the Indian Thar-Dessert [1]. It belongs to the order of *Sebacinaceous* (Basidiomycota) [2] and yields a growth promotion effect with various crop plants such as barley, tobacco, maize, and tomato, but also with the model plant *Arabidopsis thaliana* [3]. Previous studies showed that *P. indica* promotes nutrient uptake and helps plants to survive under biotic (pathogenic organisms) [4,5] and abiotic (water, temperature, salt, toxins, heavy metal ions) stress conditions [6,7]. Furthermore, it stimulates plant growth, biomass, and seed production [8,9]. The fungus colonizes the epidermal and rhizodermal part of the roots and forms pearshaped spores, which accumulate within the roots and on the root surface. *P. indica* grows inter- and intracellularly [10] but does not invade the endodermis and aerial parts of the plant. This endosymbiotic interaction has been considered as mutualistic, as it leads to an improved nutrient state in the host [11]. After establishment of this endosymbiotic interaction, the plant obtains more phosphorous and water through extracellular hyphae of the fungus, whereas the fungus is supplied with nitrogen and hydrocarbons in form of plant amino acids [11,12,13,14,15]. 

*P. indica* can be cultivated with the model plant *A. thaliana*. In general, *P. indica* colonization is host-dependent and occurs in two phases: Early interactions are biotrophic in barley and *A. thaliana*, but can switch to saprotrophy or maintain biotrophy in later stages, respectively [15]. Host metabolism determines the availability of nitrogen, and the subsequent induction of nitrogen transporters and a possible nutritional switch of *P. indica* from biotrophy to saprotrophy. *A. thaliana* had been shown to provide sufficient nitrogen sources in form of increased levels of amino acids like Gln and Asn at 14 dpi. 

During the initial phase of this interaction, defense genes are activated and reactive oxygen species (ROS) produced by the host against *P. indica* [16]. However, *P. indica* can rescue plants with elevated ROS levels by providing antioxidants [17]. After recognition of *A. thaliana*, *P. indica* releases effectors into the rhizosphere, which induce a response in the host [18]. Moreover, an increase in the intracellular calcium concentration in the host’s root cells is provoked, which triggers an intracellular signaling cascade (mitogen-activated protein kinase signaling pathways) [19,20], whereupon ethylene signaling components and ethylene-response transcription factors are required [21,22]. Furthermore, cytokinins and auxins play a crucial role in the maintenance of this symbiotic interaction [23]. Lahrmann et al. [24] and others showed that the colonization of *A. thaliana* with *P. indica* correlates with the induction of salicylic acid catabolites and jasmonate as well as glucosinolate metabolism [25,26]. Indolics were identified as key players in the maintenance of this mutualistic interaction. Especially indolic glucosinolates and reaction products are required to restrict the growth of *P. indica*. 

Since the genomes of both organisms have been completely sequenced, both partners offer an ideal opportunity to study mutualistic interactions of plants and root endophytes in the rhizosphere [27,28]. Thus, we investigated the metabolic response of *A. thaliana* to *P. indica* under hydroponic conditions by non-targeted liquid chromatography/mass spectrometry (LC/MS) and gas chromatography/mass spectrometry (GC/MS)-based metabolite profiling. For this purpose, we chose ultra performance liquid chromatography coupled to electrospray ionization quadrupole time-of-flight mass spectrometry (UPLC/ESI-QTOFMS) for the profiling of secondary metabolites and gas chromatography coupled to electron ionization quadrupole mass spectrometry (GC/EI-QMS) for the profiling of primary metabolites. Both platforms gain a snapshot of biochemical processes within a cell. Whereas reversed-phase LC/MS allows for the profiling of semipolar compounds [29], namely indolics, flavonoids, phenylpropanoids, glucosinolates and their degradation products, GC/EI-QMS covers main parts of central carbon metabolism [30]. Regardless of the choice of analysis platform, all samples can be grouped according to their common metabolic fingerprint. For this purpose we set up a standardized co-cultivation system, which supports the growth of both partners in close association to each other and the consequent profiling of roots and their exudates as well as leaves. 

## 2. Results and Discussion

To study the interaction of *P. indica* with *A. thaliana*, a sterile hydroponic cultivation system was developed, which allows for the simultaneous profiling of roots and their exudates (Supplementary Figure S1). For this purpose, *P. indica* was precultivated on agar plates and *A. thaliana* on agar-filled, bottom-cut PCR tubes protruding into a liquid culture medium. After two weeks, both organisms were brought together in half-strength Murashige-Skoog (MS) medium supplemented with 0.5% sucrose (*w*/*v*) and Gamborg B5 vitamins such as myo-inositol, nicotinic acid, pyridoxin, and thiamine. According to our preliminary studies both components are essential for this symbiosis and hence the growth promotion effect of *A. thaliana*. 

### 2.1. P. indica Promotes Shoot Growth of A. thaliana under Specific Culturing Conditions in a Hydroponic System after Root Colonization

**Figure 1:**
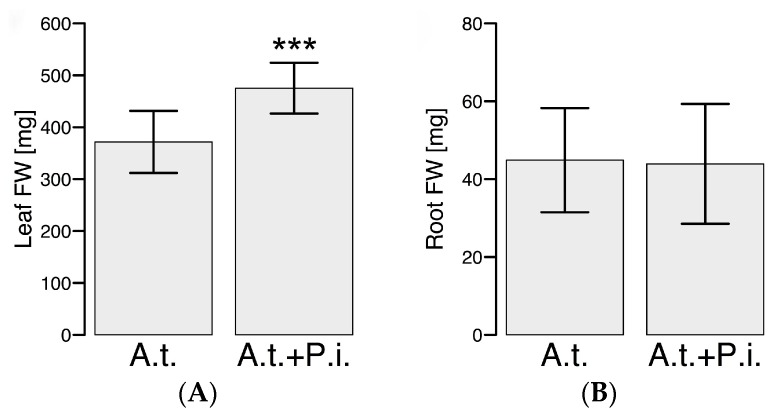
Leaf and root fresh weight of *A. thaliana* (A.t.) after co-cultivation with *P. indica* (P.i.) in a hydroponic system. *A. thaliana* was co-cultivated for two weeks with an agar plug containing mycelia of *P. indica*. For control *A. thaliana* was solely cultivated with an agar plug in 0.5× Murashige & Skoog (MS) medium supplemented with 0.5% sucrose (*w*/*v*) and vitamins: (**A**) shoot fresh weight (FW); (**B**) root fresh weight (FW). Values represent the mean ± SD (standard deviation) of three independent experiments (control samples: *n* = 3 × (3 − 5) and co-cultivated samples: *n* = 3 × 5). Each replicate n comprises a pool of 24 plants. Significance analysis of differences between control and co-cultivated samples was performed by *t*-test: ***, *p* ≤ 0.001.

If both components (sucrose and Gamborg B5 vitamins) were supplied for the co-cultivation studies, the shoot biomass increased by 22% (*p* = 4.2 × 10^−5^) in inoculated samples compared to the control confirming the previously reported growth promotion effect in soil [31]. Although *P. indica* colonizes the roots, the root biomass did not change after two weeks of co-cultivation (Figure 1) leading to the assumption that *P. indica* might provoke a systemic effect in *A. thaliana*. Although previous studies have shown a growth promotion effect in roots [23,31], we anticipated slight deviations in a hydroponic system compared to soil due to different physicochemical properties. 

To investigate how *P. indica* interacts with the host in a hydroponic system, fluorescence microscopy images were recorded using green fluorescent protein (GFP)-labeled *P. indica* and the interaction monitored at 14 dpi.

**Figure 2:**
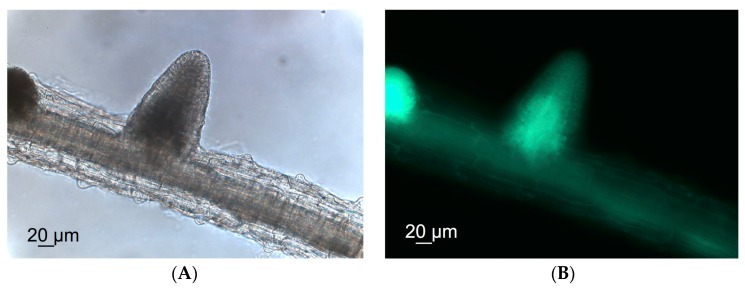
Microscopic image of an inoculated root with a GFP-labeled *P. indica* strain. (**A**) brightfield image; (**B**) fluorescence image.

*P. indica* grows inter- and intracellularly in root cells of *A. thaliana* when co-cultivated in soil [31]. In order to test if *P. indica* still forms fungal hyphae at the root surface under hydroponic conditions, a GFP-labeled *P. indica* strain was used to visualize colonization. Only weak autofluorescence signals were detected in the non-inoculated roots and roots inoculated with the non-labeled *P. indica* strain (Supplementary Figure S2). In contrast, roots inoculated with the GFP-labeled strain exhibited a very strong fluorescence already after a 3 s exposure time showing that *P. indica* colonizes the root surface and penetrates the root of *A. thaliana* (Figure 2). Interestingly, *P. indica* was predominantly detected in lateral roots. According to these observations, we concluded that *P. indica* colonizes roots of *A. thaliana* and as a consequence likely leads to changes in root and shoot metabolism. So far, only indolic glucosinolates and hormones have been discussed in depth [24,26]. 

### 2.2. P. indica Alters the Exudation of Secondary Metabolites by A. thaliana Roots

Hormonal regulation has been described to accompany the colonization of *P. indica* on *A. thaliana* roots [22,23,24,32,33,34,35]. An enrichment analysis (Table 1 and Supplementary Table S1) of the upregulated root transcripts 14 dpi as published in Lahrmann et al. [24] revealed an overrepresentation of genes involved in the Kyoto Encyclopedia of Genes and Genomes (KEGG) pathway “Biosynthesis of plant hormones” (ath01070). 

As shown in Supplementary Figure S3, *P. indica* significantly affects phytohormone levels in root exudates and roots, respectively. A higher concentration of hormones was found in exudates of co-cultivated samples as compared to control samples. This effect was in particular pronounced for jasmonate (JA), and jasmonyl-isoleucine (JA-Ile), both showing a more than 10-fold increase in the exudates and its potential role was discussed in reference [24]. In roots, the hormone content was also increased, but to a lower extent for JA, and JA-Ile, for which only a two- to four-fold increase was observed. 12-oxo-phytodienoic acid (OPDA), the precursor of JA and JA-Ile, also accumulated in roots but could not be detected in exudates irrespective of the conditions.

**Figure 3:**
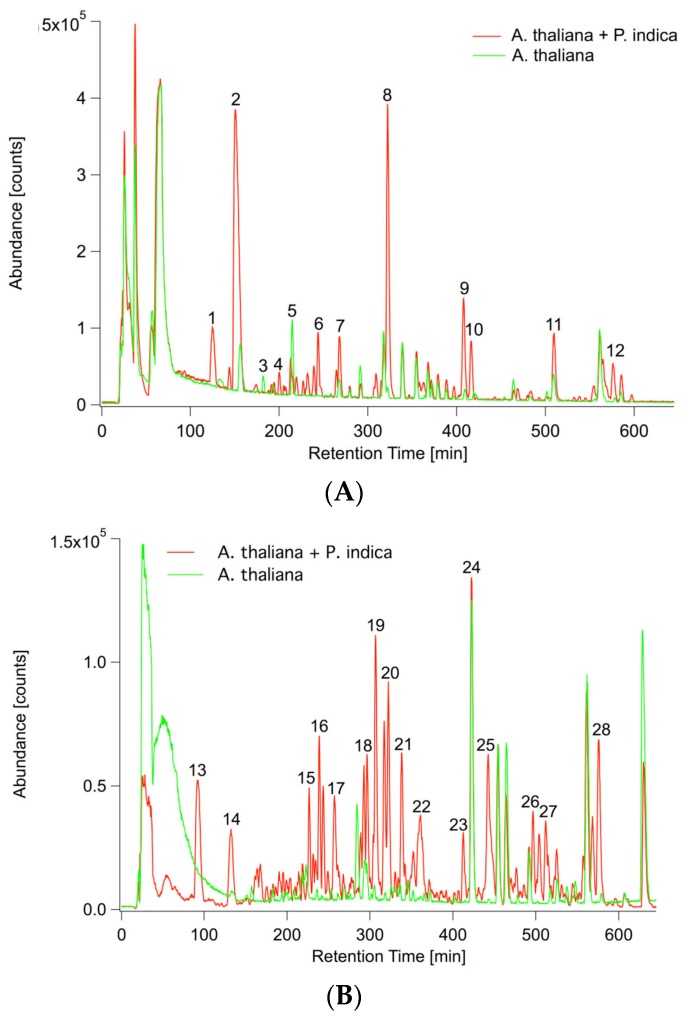
Overlay of representative UPLC/ESI(+/−)-QTOFMS base peak chromatograms (*m*/*z* 100–1000) of inoculated (red) and non-inoculated (green) *A. thaliana* exudates. (**A**) ESI(+): positive ionization mode; (**B**) ESI(−): negative ionization mode. **1**: 8-MeSO–Octyl–NH_2_; **2**: C_10_H_15_N_3_; **3**: H–Ile–Ile–OH; **4**: 1-MeO–I3CH_2_NH_2_; **5**: C_9_H_7_N_3_O_3_; **6**: C_17_H_34_NO_9_P; **7**: Scopoletin; **8**: 8-MeSO–Octyl–CN; **9**: C_16_H_29_NO_8_; **10**: C_12_H_20_O_4_; **11**: C_14_H_28_O_5_; **12**: C_28_H_42_O_6_; **13**: Pantothenic acid; **14**: C_16_H_26_O_8_; **15**: C_16_H_23_N_3_O_8_; **16**: C_16_H_23_N_3_O_8_; **17**: C_13_H_24_O_6_; **18**: C_12_H_22_O_5_; **19**: C_9_H_18_O_4_; **20**: C_13_H_22_O_5_; **21**: C_12_H_22_O_5_; **22**: C_25_H_41_N_3_O_9_; **23**: C_14_H_26_O_5_; **24**: Internal standard 2,4-Dichlorophenoxyacetic acid; **25**: 9,12,13-Trihydroxyoctadec-10-enoic acid; **26**: C_12_H_18_O_4_; **27**: C_28_H_44_O_6_; **28**: C_28_H_42_O_6_.

Besides the transcriptional regulation of hormone biosynthesis, hormone responses were overrepresented biological processes in Gene Ontology (GO:0009753 response to jasmonic acid stimulus, GO:0009751 response to salicylic acid stimulus). The analysis of Gene Ontology (GO) terms (Supplementary Table S1) further pointed to the involvement of secondary metabolic processes as the top two enriched processes (GO:0019748) ranked after defense response (GO:0006952). Consequently, roots and their exudates were comprehensively profiled for changes in primary and secondary metabolism upon *P. indica* colonization. Root exudates were only profiled for changes in semipolar metabolism, as in a screen for primary metabolites (GC/MS) all blank samples (samples without plant and/or fungus) already exhibited a considerable number of primary metabolites. Representative base peak chromatograms are depicted in Figure 3 and reveal a unique metabolic fingerprint for both ionization modes, ESI(+) and ESI(−). A principal component analysis (PCA) could verify this assumption. For both ionization modes 89% of the total variance was explained by the first principal component (PC1) and 3% ESI(+) as well as 4% ESI(−) by PC2 (Supplementary Figure S4).

Non-targeted UPLC/ESI(+/−)-QTOFMS-based metabolite profiling revealed that the concentration of 200 out of 341 detected ESI(+) components as well as 271 out of 377 ESI(−) components was significantly affected (*p* < 0.01) due to the presence of *P. indica*. A total of 28 (ESI(+)) as well as 24 (ESI(−)) components were down- and 172 (ESI(+)) as well as 247 (ESI(−)) components were upregulated due to the inoculation implying that *P. indica* stimulates root exudation of *A. thaliana*. 

**Figure 4:**
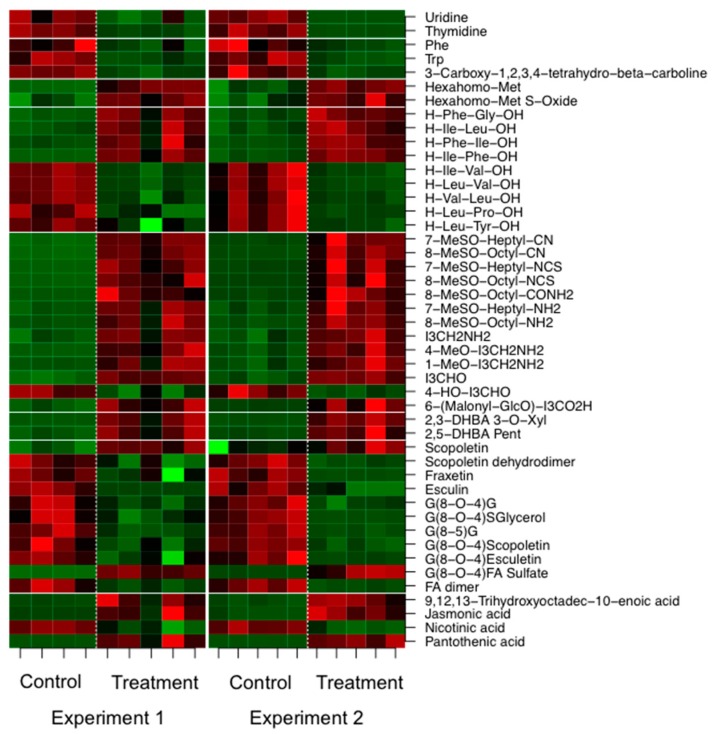
Differentially expressed metabolites (*p* ≤ 0.01) in root exudates of *A. thaliana* after co-cultivation with the fungus *P. indica* for two weeks across two independent biological experiments. Intensity values were log-transformed and z-scored row-wise. Red: maximal intensity; Green: minimal intensity.

As already observed by Lahrmann et al. [24], the amount of compounds associated with nucleoside and aromatic amino acid metabolism was reduced in concentration by the inoculation, while that of aliphatic and indolic glucosinolate metabolism (except for 4-hydroxy-indole-3-carbaldehyde), dihydroxybenzoic acid (DHBA) conjugates, JA metabolism as well as fatty acid and pantothenic acid metabolism was increased. A number of phenylpropanoids including coumarins and oligolignols (except for scopoletin and G(8-*O*-4)FA sulfate) showed reduced levels in the exudates of inoculated samples (Figure 4) leading to the assumption that these oligomers are further metabolized inside the cell and not exuded, very likely to oligolignols or to lignin [36], a main constituent of the cell wall. Both, glucosinolate (ath00966) and phenylpropanoid biosynthesis (ath00940), were among the overrepresented KEGG pathways of root transcripts (Table 1).

Nicotinic acid, an important precursor for vitamin B6, and thus, key player in the photoprotection of plants [37], also decreased in concentration upon co-cultivation in the root exudates (Figure 4). Obviously, nicotinic acid is required by *P. indica*. If this compound was not supplemented, no growth-promoting effect was observed of the host. 

In the exudates we also detected differences in the dipeptide pool, namely the concentration of Phe-Gly, Ile-Leu, Phe-Ile and Ile-Phe was enhanced, while that of Ile-Val, Leu-Val, Val-Leu, Leu-Pro and Leu-Tyr was reduced in the co-cultivated samples (Figure 4). These differences might originate from different functionalities of the respective dipeptides and require further investigation. So far, Komarova et al. [38] showed that peptide transporters (*AtPTR5* and *AtPTR5*) facilitate the uptake of nitrogen from the rhizosphere.

### 2.3. Changes in the Root Metabolism of A. thaliana

The secondary metabolic changes detected in root exudates, especially that of glucosinolate biosynthesis, phenylpropanoid biosynthesis, and phenylalanine metabolism should also be reflected in root metabolism. In addition, transcriptionally enriched KEGG pathways of primary metabolism (Table 1), such as nitrogen metabolism (ath00910), glycine, serine and threonine metabolism (ath00260), and cyanoamino acid metabolism (ath00460) were expected in the GC/MS-based metabolite profiles. 

#### 2.3.1. Non-Targeted GC/MS Based Metabolite Profiling Reveals Perturbations in the Primary Root Metabolism

**Figure 5:**
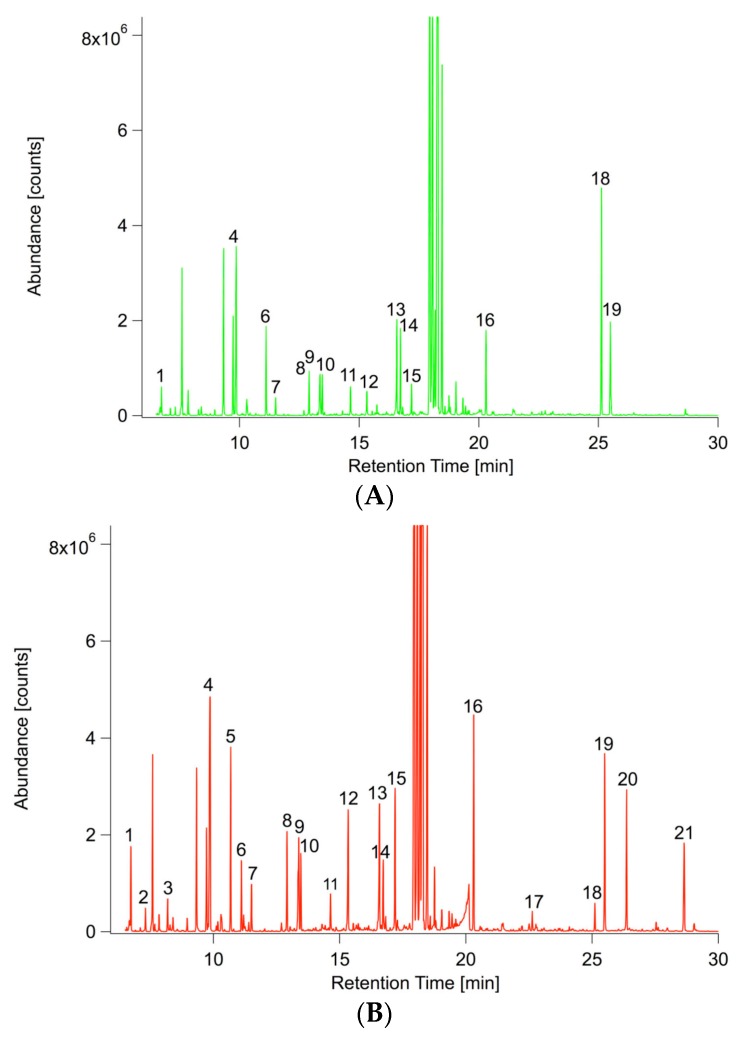
Representative extracted ion chromatograms (*m*/*z* 73) of inoculated and non-inoculated *A. thaliana* root extracts. (**A**) Non-inoculated root; (**B**) with *P. indica* inoculated root. **1**: Lactic acid (2TMS); **2**: Alanine (2TMS); **3**: Sulfuric acid (2TMS); **4**: Phosphoric acid (3TMS); **5**: Glyceric acid (3TMS); **6**: Serine (3TMS); **7**: Threonine (3TMS); **8**: Malic acid (3TMS); **9**: Pyroglutamic acid (2TMS); **10**: GABA (3TMS); **11**: Glutamic acid (3TMS); **12**: Asparagine (3TMS); **13**: Glutamic acid (3TMS); **14**: Glutamine (3TMS); **15**: Citric acid (4TMS); **16**: Myo-Inositol (6TMS); **17**: Glucose-6-phosphate (1MeOX, 6TMS); **18**: Thiamine hexoside; **19**: Sucrose (8TMS); **20**: Unknown; **21**: Unknown.

Figure 5 shows two representative extracted ion chromatograms of *m*/*z* 73 (equals C_3_H_9_Si^+^ and is a typical fragment for trimethylsilylated compounds) obtained from a pool of inoculated and non-inoculated roots. Again, the inoculated profile is distinct compared to the non-inoculated root metabolic profile. Forty-eight percent of the total variance was explained by PC1 and 13% by PC2 and are plotted in Supplementary Figure S5.

**Figure 6:**
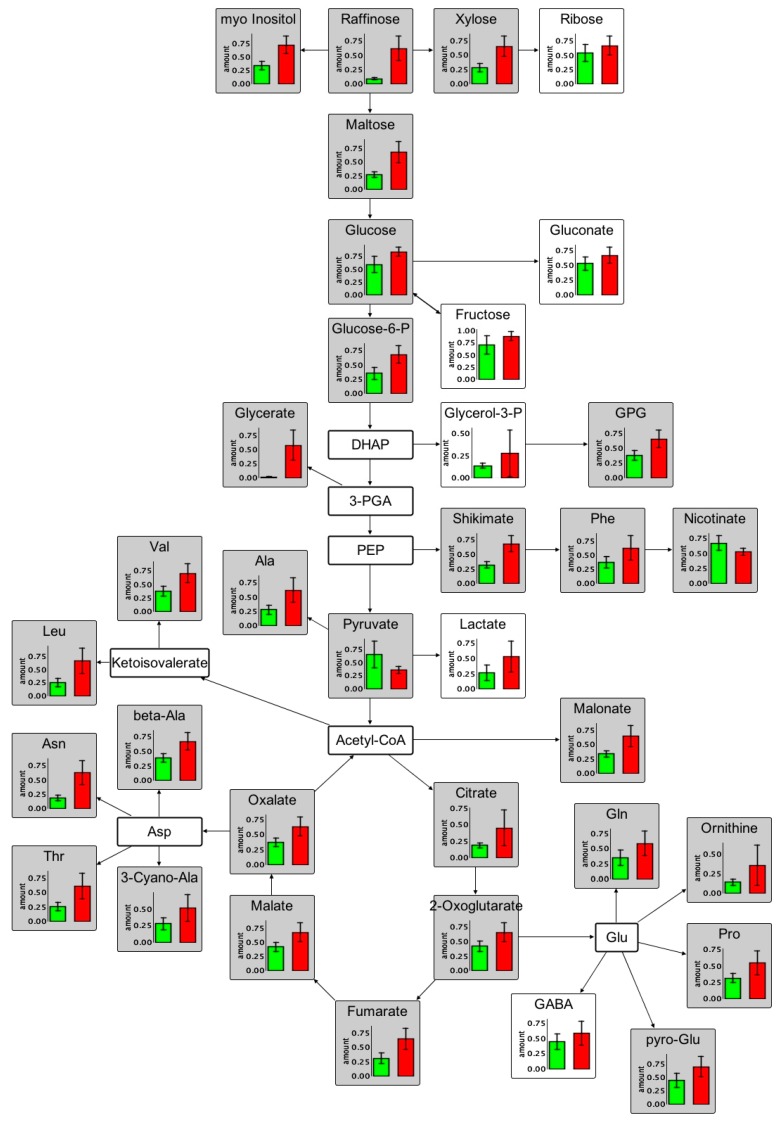

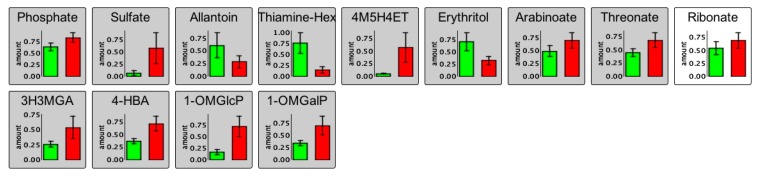
Differentially expressed primary metabolites occurring in root extracts of *A. thaliana*. Control and treatments are color-coded; control: *A. thaliana* (green) and treatment: *A. thaliana* + *P. indica* (red). Compounds with *p* < 0.01 are specifically marked by grey color or left blank for 0.01 ≤ *p* ≤ 0.05. GPG: glycerophosphoglycerol; 4M5HET: 4-methyl-5-hydroxyethylthiazole; 3H3MGA: 3-hydroxy-3-methylglutaric acid; 4-HBA: 4-hydroxybenzoic acid; 1-OMGclP: 1-*O*-methyl-glucopyranoside; 1-OMGalP: 1-*O*-methylgalactopyranoside.

Non-targeted GC/EI-Q-MS based metabolite profiling revealed 287 out of 801 differentially accumulated components. Among them, we detected amino acids (e.g., Asn, Thr, Leu, 3-Cyano-Ala, beta-Ala, Val, Ala, Gln, ornithine, Pro, pyro-Glu, and GABA), organic acids (e.g., citrate, 2-oxoglutarate, fumarate, malate, oxalate, glycerate, fumarate, and 3-hydroxy-3-methylglutaric acid), carbohydrates (e.g., 1-*O*-methylglucopyranoside, 1-*O*-methylgalactopyraoside, maltose, raffinose, trehalose, xylose, ribose), polyols (erythritol, myo-inositol), phosphates (e.g., glycerol-3-phosphate, phosphate, glycerophosphoglycerol), and sulfates (e.g., sulfate, thiamine, thiamine-hex) belonging to the starch and sucrose metabolism, glycolysis, tricarboxylic acid (TCA) cycle, amino acid metabolism, and urea metabolism. All compounds showed increased levels in the inoculated roots (Figure 6) except for pyruvate, erythritol, allantoin, and 4-methyl-5-thiazoleethanolglycopyranoside (for spectrum see Supplementary Figure S6) indicating that the initially applied amount of sucrose and thiamine is metabolized by *P. indica*. We observed an increase in the concentration of Asn, Gln, Ser, Thr, and Ala at 14 dpi. Serine was also increased in its levels as described by Lahrmann et al. [15], but did not pass the defined significance level (*p* = 0.051). In general, the data collected are in good accordance with the transcriptional changes and lead us to the hypothesis that *A. thaliana* provides nitrogen to the fungus so that *P. indica* can maintain a biotrophic nutritional state [15]. In the leaf profiles, the N-rich amino acids (Gln, Arg, Asn, 3-Cyano-Ala, and ornithine) were among the few differentially accumulated compounds decreasing in concentration upon colonization and consequently showed the opposite trend (Supplementary Figure S7) compared to the roots. This raises the question if these amino acids are transported to the root to feed *P. indica*. Most likely, these amino acids are required to balance the nutritional state of *P. indica*. To trace the flow of nutrients, further investigations are required. The change in the concentration of organic acids and carbohydrates was comparable for roots and leaves except that less differential changes were observed in the leaf profiles. These results again show that *P. indica* activates primary root metabolism of *A. thaliana*. According to our data, both partners appear to offer each other nutrients to maintain a mutualistic interaction, since an enhanced amount of P, S, and N (in the form of amino acids) was observed in roots of *A. thaliana* colonized with *P. indica*. 

#### 2.3.2. LC/MS Based Non-Targeted Metabolite Profiling Shows an Induction of Aliphatic and Indolic Glucosinolate Metabolism, Flavonoids, and Oligolignols in Roots

**Figure 7:**
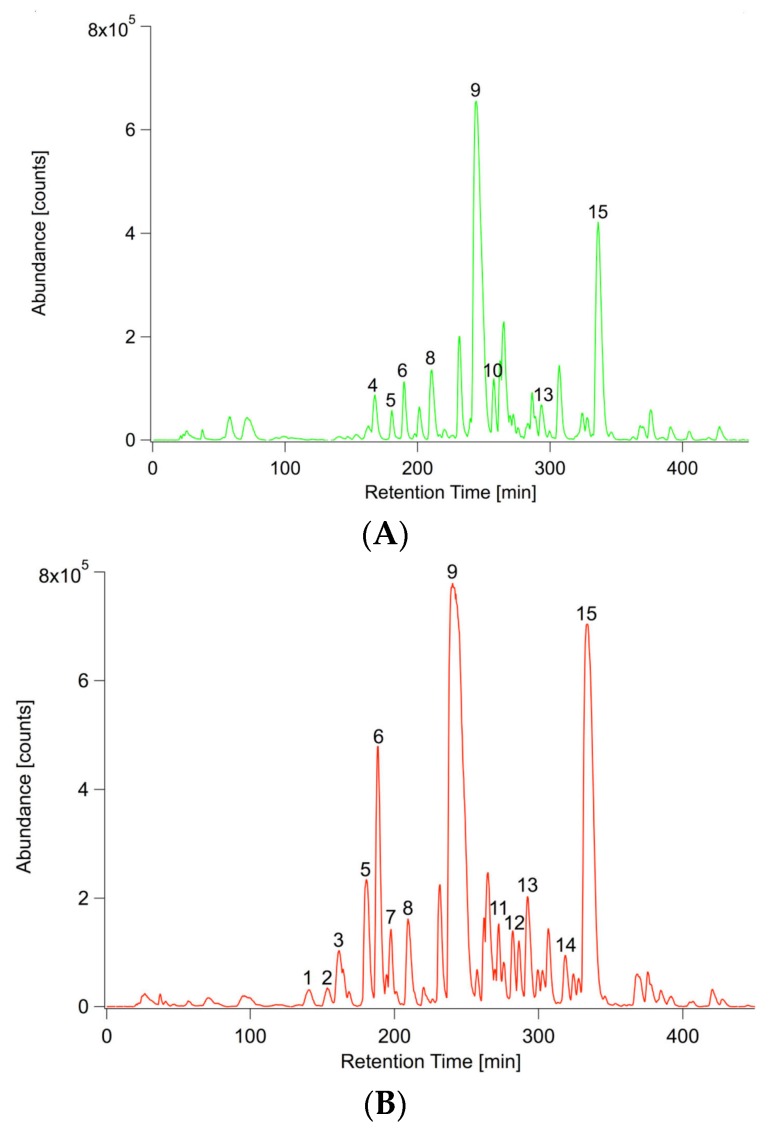
Representative UPLC/ESI(−)-QTOFMS base peak chromatograms (*m*/*z* 100–1000) of inoculated and non-inoculated *A. thaliana* root extracts. (**A**) Non-inoculated root; (**B**) with *P. indica* inoculated root. **1**: 7MeSO Heptyl GSL; **2**: 2,5 DHBA-Pent; **3**: I3M GSL; **4**: C_14_H_18_O_10_; **5**: C_17_H_24_O_10_; **6**: 8 MeSO Octyl GSL; **7**: Scopolin; **8**: 4MeO-I3M GSL **9**: 1MeO-I3M GSL; **10**: C_18_H_32_O_11_; **11**: C_19_H_18_O_3_; **12**: C_19_H1_8_O_3_; **13**: 7MeS Heptyl GSL; **14**: C_38_H_46_O_18_; **15**: 8MeS Octyl GSL.

Besides primary metabolism, secondary root metabolism was investigated, since one category “secondary metabolic process” was a highly ranked candidate in the GO enrichment analysis. A unique fingerprint was observed in the root LC/MS profiles (Figure 7). According to Supplementary Figure S5, 76% of the entire variance was explained by PC1 and 0.07% by PC2 for the positive mode. These values were similar for the negative mode (PC1: 74%; PC2: 0.08%) and indicate that secondary metabolism is perturbed to a greater extent than primary metabolism. 

In these profiles, 167 out of 329 detected compounds (ESI(+)) were altered in abundance and 188 out of 359 for the negative ionization mode due to the presence of *P. indica*. Similarly to the exudates, a higher number of compounds displayed upregulated abundance in the inoculated samples compared to the non-inoculated samples. From these numbers one can once more conclude that *P. indica* stimulates secondary root metabolism as well. 

**Figure 8:**
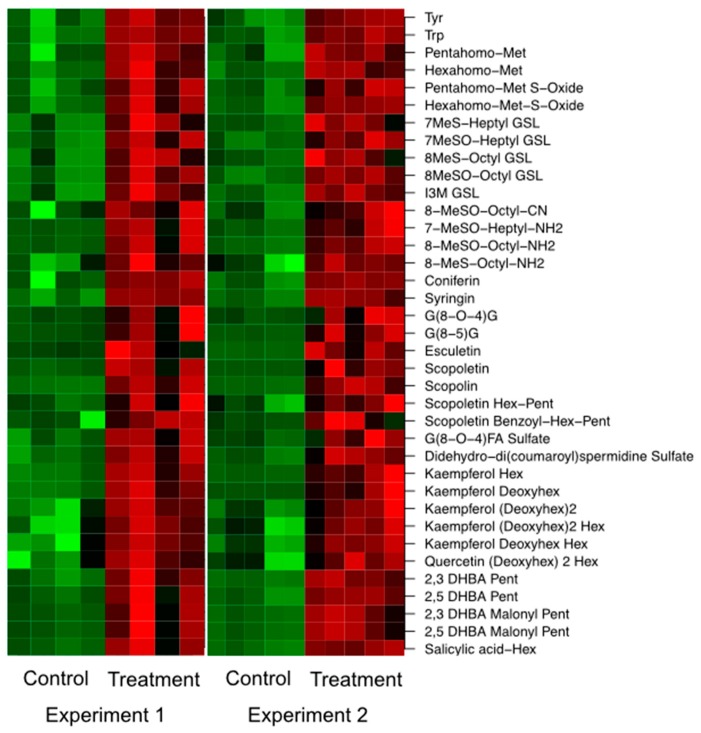
Differentially expressed secondary metabolites occurring in root extracts of *A. thaliana* across two independent biological experiments. Candidates were retrieved from a two-sided *t*-test (*p* < 0.01). For visualization, intensity values were log-transformed and z-scored row-wise. Red: maximal intensity; green: minimal intensity.

In accordance with Lahrmann et al. [24], aliphatic and indolic glucosinolates as well as their breakdown products, aromatic amino acids, coumarins, oligolignols, and flavonoids accumulated in inoculated roots (Figure 8) confirming the transcript data (KEGG, Table 1: glucosinolate ath00966 and phenylpropanoid biosynthesis ath00940). Although the plant seems to be in a defensive stage, no camalexin was detected in these profiles. In the leaf profiles an increased amount of aliphatic and indolic glucosinolates as well as their breakdown products, JA conjugates, oligolignols, and hydroxycinnamic acid amides was detected (Supplementary Figure S8). Several flavonoids (glycosylated kaempferol and quercetin) were only detected as differential in the root profiles and not in the leaf profiles, leading to the conclusion that this substance class plays an important role in the mutualistic interaction of *A. thaliana* and *P. indica*. Recently, Lahrmann et al. [24] stated that it remains to be clarified if flavonoids are accumulating in roots of *A. thaliana* upon interaction with *P. indica*. Indeed, we show that flavonoids accumulate in roots of *A. thaliana* upon co-cultivation with *P. indica*. Most likely, enhanced flavonoid biosynthesis, in addition to JA signaling [39], may also function as a signal for *P. indica*.

## 3. Materials and Methods

### 3.1. Chemicals and Standards

All chemicals were of highest analytical grade (>99%) and obtained from Carl Roth GmbH + Co. KG (Karlsruhe, Germany), Difco Microbiology (Lawrence, KS, USA), Duchefa Biochemie B.V. (Haarlem, The Netherlands), Merck KGaA (Darmstadt, Germany), and Sigma-Aldrich (Steinheim, Germany).

### 3.2. Pre-Cultivation of P. indica

*P. indica* was cultured on agar plates (1.5% (*w*/*v*) agar) for 3 weeks at 28 °C in the dark using Aspergillus minimal medium [29]. For this purpose, a punched out agar block with mycelia of *P. indica* was placed in the center of a culture plate. 

### 3.3. Conduction of Co-Cultivation Studies and Production of Plant Material

Co-cultivation studies were performed as previously described [25]. In short, two-week old *A. thaliana* plantlets were co-cultivated for two weeks with *P. indica* in a hydroponic system under short day conditions (23 °C, 8 h light, 180 μE·m^−2^·s^−1^ and 21 °C, 16 h dark). After two weeks of co-cultivation (four-week old plants), the medium containing the nutrient solution and the root exudates was filtered and stored at 4 °C in Schott flasks until further processing. At harvest, roots were cut below the bottom of the PCR tube and blotted dry with a paper towel before shock freezing in liquid nitrogen. Finally, they were stored at −80 °C until further processing. More technical details are visualized in Supplementary Figure S1. Media composition is summarized in Appendix A.

### 3.4. LC/MS-Based Metabolite Profiling

For LC/MS-based metabolite profiling (**UPLC**: Acquity, Waters, Eschborn, Germany; **MS**: MicrOTOF–Q I hybrid quadrupole time-of-flight mass spectrometer equipped with an Apollo II electrospray ion source, Bruker Daltonik GmbH, Bremen, Germany), the ground tissue material was processed by solid liquid extraction using methanol/water, 80/20 (*v*/*v*) (40 mg root fresh weight corresponds to 200 µL extraction solution and 50 mg leaf fresh weight corresponds to 400 µL extraction solution). Analytes of the nutrient solution were extracted by a reversed-phase solid phase extraction procedure (180 mL medium result in 120 µL analysis solution).

#### 3.4.1. Preparation of Nutrient Solutions for LC/MS Analysis

All exudate samples were prepared and analyzed by UPLC/ESI-QTOFMS as presented in Lahrmann et al. [24]. In short, the nutrient solution was spiked with 20 µM 2-(2,4-dichlorophenoxy)acetic acid, evaporated until dryness, reconstituted in 9 mL water/methanol 95/5 (*v*/*v*) and subjected to a Bond Elut PPL cartridge (200 mg, 3 mL, Agilent Technologies, Böblingen, Germany). Finally, the eluate was subjected to a solid phase extraction workup and reconstituted in 120 µL water/methanol 70/30 (*v*/*v*) prior to LC/MS analysis. Technical details of the solid phase extraction workup can be found in the Appendix B.

#### 3.4.2. Sample Preparation and Profiling of Tissue Material for LC/MS Analysis

The plant material was processed according to Böttcher et al. [29]. As already described, the frozen material was extracted twice with methanol/water, 80/20 (*v*/*v*) and reconstituted in methanol/water, 30/70 (*v*/*v*) prior to LC/MS-analysis. More details of the extraction procedure can be found in the Appendix B.

#### 3.4.3. Non-Targeted LC/MS-Based Profiling and Data Analysis

Changes in the secondary plant metabolism were analyzed by UPLC/ESI-QTOFMS. Samples were injected onto an Acquity UPLC system (Waters, Eschborn, Germany), equipped with an HSS T3 column (100 × 1.0 mm, particle size 1.8 µm, Waters), and separated using a binary gradient (A: water/0.1% (*v*/*v*) formic acid; B: acetonitrile/0.1% (*v*/*v*) formic acid). Eluting compounds were detected in positive and negative ionization mode from *m*/*z* 100–1000 using a MicroTOF–Q I hybrid quadrupole time-of-flight mass spectrometer equipped with an Apollo II electrospray ion source (Bruker Daltonics, Billerica, MA, USA). All instrument parameters and further settings can be found in the Appendix B. 

Raw data files were converted to mzData using CompassXPort version 1.3.10 (Bruker Daltonics). For feature detection, alignment, and filling of missing values the R package XCMS version 1.41.0 [40] was used. Settings are summarized in Appendix B. 

The intensities of the resulting features (*m*/*z*-retention time pairs) were log_2_ transformed and subjected to a two-sided Student’s *t*-test. Relevant mass spectral features were extracted within a predefined range (isolation width: ±0.02 *m*/*z*) and elemental compositions were calculated applying a default error range (15 ppm). Putative elemental compositions were checked for consistency while analyzing elemental compositions of fragment ions and neutral losses of collision-induced dissociation (CID)-mass spectra. For acquisition of CID mass spectra quasi-molecular cluster ions were isolated at the Q1 (isolation width: ±3 *m*/*z*) and fragmented inside the collision cell using argon as collision gas. Product ions were detected as described above. All mass spectral data can be found in the MetaboLights repository (MTBLS341) [41]. 

### 3.5. GC/MS Based Metabolite Profiling

#### 3.5.1. Sample Preparation of Tissue Material

One hundred µL extract of the remainder from the LC/MS-based metabolite profiling studies was spiked with 100 µM succinic acid-2,2,3,3-d_4_, dried down in a vacuum concentrator, and stored at −20 °C until further processing.

#### 3.5.2. Preparation of Samples for Non-Targeted Metabolite Profiling and Analysis of GC/MS Profiles

Dried down extracts were subjected to a two-step derivatization process using methoxyamine hydrochloride and *N*,*O*-bis(trimethylsilyl)trifluoroacetamide. Derivatized samples were injected splitless at 230 °C onto an Agilent 6890N GC equipped with a split/splitless inlet and a ZB-5 column (30 m × 0.25 mm, 0.25 µm 95% dimethyl/5% diphenyl polysiloxane film, 10 m integrated guard column, Phenomenex, Aschaffenburg, Germany). Eluting components were detected from *m*/*z* 70–600 by using an Agilent 5975 Series Mass Selective Detector (Agilent Technologies, Waldbronn, Germany). For the generation of the metabolite profiles, chromatograms were baseline-corrected using Metalign [42]. Peak intensities above 500 arbitrary ion current units were imported into the TagFinder software [43], aligned using the retention index model of van den Dool and grouped according to their common retention time and mass spectral features. For statistical analysis, peak intensities of cluster (cluster size > 3) were normalized to the internal standard (succinic acid-2,2,3,3-d_4_). Then, all data were log_2_-transformed and submitted to a two-sided Students *t*-test. Finally, resulting mass spectral features were identified via best mass spectral and retention index match using the Golm Metabolome Database [44] and the NIST2012 software (May 2011, National Institute of Standards and Technology, Gaithersburg, MD, USA). Details of the derivatization protocol and instrument parameters can be found in the Appendix C.

All statistical analysis was either performed with the R statistical language, the Bioconductor environment, the package pcaMethods or Microsoft Excel.

### 3.6. Hormone Analysis

Hormone profiling was conducted as described in Ziegler et al. [45] (for further information see Appendix D). Root material was homogenized, extracted in methanol, and processed firstly using a hydrophobic solid phase extraction cartridge (Chromabond Sorbent HR-XC, Macherey-Nagel, Düren, Germany) and secondly with an anion exchange solid phase extraction cartridge (Diethylaminoethyl Sephadex (DEAE-Sephadex)). For the root exudates the anion exchange step was omitted. 

Analytes were separated by an Agilent 1290 Infinity HPLC system and detected on-line by ESI-MS/MS using an API 3200 triple-quadrupole LC-MS/MS system equipped with an ESI Turbo Ion Spray interface (AB Sciex, Darmstadt, Germany). Triple quadrupole scans were acquired in the multiple reaction monitoring mode (MRM) with Q1 and Q3 set at unit resolution. Scheduled MRM was performed with a window of 90 s and a target scan time of 0.1 s. Selected MRM transitions and compound specific parameters can be found in Ziegler et al. [45].

### 3.7. Microscope Images

Bright-field and fluorescence microscopic images were recorded with a Stemi 2000 Axio Imager stereomicroscope (Carl Zeiss MicroImaging GmbH, Göttingen, Germany). For bright-field images a Plan Apochromat 20×/0.75 objective with 20× magnification was used and for fluorescence images a Plan Apochromat 20×/0.75 objective with 20× magnification, a GFP-Filter 450–490 nm, filterset 9 and the Axio Imager camera. 

### 3.8. Transcript Enrichment Analysis

Overrepresentation analysis of the overexpressed genes in *Arabidopsis* 14 dpi as published in Lahrmann et al. [24] was performed with DAVID [46,47] against the default background genes from TAIR using KEGG pathways [48] and Gene Ontology [49]. 

### 3.9. Data Availability

All data sets are available from the MetaboLights repository [41] under the accession number MTBLS341.

## 4. Conclusions

The mutualistic interaction of *P. indica* with *A. thaliana* resulted in an increased shoot biomass production, but not root biomass after a two-week co-cultivation. Interestingly, the presence of *P. indica* had an obvious effect on the root’s primary and secondary metabolism and the exudation rate, but not on leaf metabolism of *A. thaliana*. Apparently, *P. indica* stimulates the belowground metabolism of *A. thaliana*, but not the shoot metabolism. The metabolic changes identified can be considered as potential biomarkers, which need to be tackled in the near future. Previous studies and this study have shown that indolic glucosinolates and hormones are important for the interaction. The induction of the defense response might indicate that the plant tries to balance fungal growth and maintain its mutualism. This assumption could be confirmed by the analysis of appropriate mutants. In the future, new mutants, especially of the flavonoid metabolism, need to be obtained to investigate the mutualistic interaction in more depth. It is possible that plant-growth promoting microorganisms can be valuable tools for crop improvement [7,50], as they promote the plant growth and help the plant to cope with abiotic and biotic stress factors.

## Figures and Tables

**Table 1:** Overrepresented KEGG pathways among upregulated *A. thaliana* root transcripts 14 dpi [15].

Term	Fold Enrichment	*p*-Value *
ath00966: Glucosinolate biosynthesis	10.4	8.89 × 10^−8^
ath00940: Phenylpropanoid biosynthesis	3.8	5.84 × 10^−7^
ath00360: Phenylalanine metabolism	3.7	6.21 × 10^−5^
ath00903: Limonene and pinene degradation	3.8	1.12 × 10^−4^
ath00680: Methane metabolism	3.5	1.70 × 10^−4^
ath00945: Stilbenoid, diarylheptanoid and gingerol biosynthesis	3.7	2.20 × 10^−4^
ath00910: Nitrogen metabolism	3.9	5.56 × 10^−3^
ath00260: Glycine, serine and threonine metabolism	3.7	7.07 × 10^−3^
ath00460: Cyanoamino acid metabolism	5.0	1.25 × 10^−2^
ath00960: Tropane, piperidine and pyridine alkaloid biosynthesis	5.5	2.05 × 10^−2^
ath01070: Biosynthesis of plant hormones	1.6	3.54 × 10^−2^
ath00400: Phenylalanine, tyrosine and tryptophan biosynthesis	3.3	4.44 × 10^−2^

* *p*-value was corrected according to Benjamini-Hochberg.

## Supplementary Materials

Supplementary materials can be found at http://www.mdpi.com/1422-0067/17/7/1091/s1.

## Abbreviations

*A. thaliana*	*Arabidopsis thaliana*
*P. indica*	*Piriformospora indica*
GC	Gas chromatography
UPLC	Ultraperformance liquid chromatography
ESI	Electrospray ionisation
QTOF	Quadupole time of flight mass spectrometer
SD	Standard deviation
ET	Ethylene
JA	Jasmonic acid
GSL	Glucosinolate

## Appendix A (Media for Co-Cultivation Studies)

Cultivation of *P. indica*: For the cultivation of *P. indica* Complete Medium was used and prepared as follows: *stock solution 1*: 12% (*w*/*v*) NaNO_3_, 1.04% (*w*/*v*) KCl, 1.04% (*w*/*v*) MgSO_4_·7H_2_O, 3.03% (*w*/*v*) KH_2_PO_4_ and *stock solution 2*: 0.6% (*w*/*v*) MnCl_2_·4H_2_O, 0.265% (*w*/*v*) ZnSO_4_·7H_2_O, 0.15% (*w*/*v*) H_3_BO_3_, 0.075% (*w*/*v*) KI, 0.025% (*w*/*v*) Na_2_MO_4_·2H_2_O, 0.013% (*w*/*v*) CuSO_4_·5H_2_O. The final medium consisted of a mix of 5% (*v*/*v*) *stock solution 1*, 2% (*w*/*v*) Glucose, 0.2% (*w*/*v*) Bacto-Pepton, 0.1% (*w*/*v*) yeast extract, 0.1% (*w*/*v*) Casamino acids and 0.1% (*v*/*v*) *stock solution 2*.

Cultivation of *A. thaliana* and co-cultivation of *A. thaliana* with *P. indica*: For the pre- and co-cultivation stage 0.221% (*w*/*v*) Premix (M0231; Duchefa Biochemie B.V.) and 0.5% (*w*/*v*) sucrose in water were used. The pH was adjusted to 5.9 with 1 M KOH prior to autoclaving.

## Appendix B (UPLC/ESI-QTOFMS)

C18-SPE: Bond Elut PPL cartridges were washed with 1 mL methanol, conditioned with 1 mL water/formic acid, 98/2 (*v*/*v*), loaded with 4 mL sample solution, washed with 1 mL water, and eluted with 2 mL methanol/formic acid, 99/2 (*v*/*v*); eluates were evaporated in a vacuum centrifuge and the residue were reconstituted in 120 µL water/methanol, 70/30 (*v*/*v*).

Extraction of root material: 200 μL methanol/water, 80/20 (*v*/*v*), pre-cooled at −28 °C, were added to the tissue; the mixture was allowed to reach room temperature within 15 min with occasionally vortexing; after sonication for 15 min at 20 °C and centrifugation for 10 min at 16,000× *g* the supernatant was transferred to a new 2 mL polypropylene tube; the remaining plant material was extracted a second time with 200 µL methanol/water, 80/20 (*v*/*v*); both extracts were combined and evaporated to dryness at 40 °C using a vacuum centrifuge; the residue was redissolved in methanol/water, 30/70 (*v*/*v*) according to fresh weight (40 mg = 200 µL), sonicated for 10 min at 20 °C, centrifuged for 5 min at 16,000× *g*, and the supernatant subjected to UPLC/ESI-QTOFMS analysis

UPLC settings: Full loop (loop volume: 2.5 µL); gradient: (flow rate: 150 µL·min^−1^) 0–1 min, isocratic 95% A, 5% B; 1–16 min, linear from 5% to 95% B; 16–18 min, isocratic 95% B; 18–18.01 min, linear from 95% to 5% B; 18.01–20 min, isocratic 5% B.

ESI(+) settings: Nebulizer gas, nitrogen, 1.6 bar; dry gas, nitrogen, 6 L·min^−1^, 190 °C; capillary, −5000 V; end plate offset, −500 V; funnel 1 RF, 200 Vpp; funnel 2 RF, 200 Vpp; in-source CID energy, 0 V; hexapole RF, 100 Vpp; quadrupole ion energy, 3 eV; collision gas, argon; collision energy, 3 eV; collision RF 200 Vpp; transfer time, 70 μs; pre pulse storage, 5 μs; spectra rate, 3 Hz.

ESI(−) settings: All parameters were maintained except for the nebulizer gas (1.4 bar), capillary (4000 V), quadrupole ion energy (5 eV), collision energy (7 eV), and collision RF (150 Vpp).

Data acquisition: centroid mode; recalibration on the basis of lithium formate cluster ions after injecting 20 μL 10 mM lithium hydroxide 49.9/49.9/0.2 (dissolved in isopropanol/water/formic acid; *v*/*v*/*v*).

XCMS settings: Feature detection with the help of the centWave algorithm (sntresh: 3, prefilter: (3.100), ppm: 25, peak width: (5.12); feature alignment with the help of the XCMS function group.density (minfrac: 0.75, bw: 2, mzwid: 0.05); missing values replacement by the XCMS function fillPeaks.

Analysis of raw data: DataAnalysis 4.2 software (Bruker Daltonics) for deconvolution and generation of extracted ion chromatograms

## Appendix C (GC/EI-QMS)

Derivatization: Residues were reconstituted in 40 µL methoxyaminehydrochloride (20 mg/mL in pyridine, Sigma-Aldrich), the solution thoroughly vortexed and incubated at 40 °C for 1.5 h. An 80 µL mix comprising 70 µL *N*,*O*-bis(trimethylsilyl)trifluoroacetamide (BSTFA, Macherey-Nagel) and 10 µL alkane reference mixture (dodecane, pentadecane, nonadecane, docosane, octacosane and dotriacontane each to a final concentration of 80 µg·mL^−1^ dissolved in pyridine) were added and incubation at 40 °C proceeded for an additional 30 min; the solution was centrifuged and the supernatant transferred to a GC vial.

GC settings: Carrier gas helium, constant flow: 1 mL·min^−1^; temperature program: 70 °C for 1 min, gradient 9 K·min^−1^ to 300 °C, 5 min at 300 °C.

EI settings: Transfer line 300 °C; ion source temperature 230 °C; scan rate 3 Hz

Metalign settings: Maximum amplitude: 6,000,000, peak slope factor: 0.5, peak threshold factor: 1, average peak width at half height: 5.

TagFinder settings: Peak Finder (Smooth Width Apex Finder: 3; Low Intensity Threshold: 500; Max: Merging Time Width: 0.3); Time Scanner (Time Scan Width: 1; Min Fragment Intensity: 500); Tag Gen Filter (Tag Mass: 76, 146, 150–600; Sample Counts > 5); Intensity Calculator (Simple: MAX_INTENSITY); Tag Correlation (Correlation Method: PearsonCor; Significance Level: SIG_005); Tag Clustering (Core Adjacency Option: SAME_CORE; Min Core Option: INPUT_VALUE); Tag Output (Min Cluster Size: 3)

## Appendix D (Hormone Analysis)

### Profiling of Root Tissue

Homogenization and extraction: Root material was homogenized in bead beater and extracted with; 200 µL methanol (supplemented with 2 ng abscisic acid-d_6_ (ABA-d_6_), 5 ng indole-3-acetic acid-^13^C_6_ (IAA-^13^C_6_), 5 ng jasmonic acid-d_6_ (JA-d_6_), 0.74 ng jasmonyl isoleucine-d_2_ (JA-Ile-d_2_), 30 ng 12-oxo phytodienoic acid (OPDA-d_5_), 1.5 ng salicylic acid-d_4_ (SA-d_4_), 5 ng zeatin (Z-d_5_), 5 ng trans-zeatin-riboside-d_5_ (tZ9R-d_5_), 5 ng dihydrozeatin riboside-d_5_ (DHZR-d_5_). After vortexing for 20 min the supernatant was clarified by two rounds of centrifugation at 10,000 rpm for 5 min. Before loading on the HR-XC SPE 1 mL water/acetic acid, 98/2 (*v*/*v*) was added.

HR-XC: The resin was conditioned with 1 mL methanol followed by 1 mL water (the liquid was passed through SPE 96 well plate (50 mg HR-XC resin per well) by centrifugation at 250× *g* for 5 min using a JS5.3 bucket rotor in an Avanti J-26XP centrifuge (Beckman). Samples were transferred to the SPE 96 well plate, the resin washed with 1 mL H_2_O. Analytes were eluted successively by adding 1 mL MeOH (for acidic hormones) and 1 mL methanolic ammonia (0.35 M) for zeatins.

DEAE-Sephadex: The resin was washed with 1 mL methanol. The methanolic eluates from the HR-XC plates were loaded onto DEAE-Sephadex (acetate form, 50 mg·well^−1^) filled. After washing with 1 mL methanol, the analytes were eluted with 1 mL of 3 M acetic acid in methanol.

Further processing: eluates were transferred to 2 mL Eppendorf tubes and evaporated to dryness; residues were dissolved in 40 µL of 20% (*v*/*v*) methanol, diluted with 40 μL of water and centrifuged at 10,000× *g* for 10 min.

LC: Agilent 1290 Infinity HPLC; Nucleoshell RP18 column (50 × 3 mm, particle size 2.7 μm; Macherey-Nagel, Düren, Germany) at 30 °C; eluent (A: water/0.2% (*v*/*v*) acetic acid; B: acetonitrile/0.2% (*v*/*v*) acetic acid); flow rate: 0.5 mL·min^−1^; gradient for cytokinins: 2% B for 0.5 min, followed by a linear increase to 28% B within 3 min; increase to 98% in 0.5 min followed by an isocratic period of 1.5 min at 98% B, starting conditions restored within the next 0.5 min, and the column was allowed to re-equilibrate for 1 min at 2% B; gradient for acidic phytohormones: B increased from 10% to 80% within 9 min after an initial hold at 10% B for 0.5 min; further increase to 98% B within 0.5 min; isocratic period at 98% B for 1.5 min; column re-equilibrated at 10% B for 1 min.

ESI(+) for cytokinins: curtain gas 50 psi, ion spray voltage 3500 V, ion source temperature 650 °C, nebulizing and drying gas 70 psi and 50 psi.

ESI(−) for acidic phytohormones: negative ion mode curtain gas 50 psi, ion spray voltage −4500 V, ion source temperature 350 °C, nebulizing and drying gas 70 psi and 50 psi.

Data evaluation: Peak areas were calculated automatically using the IntelliQuant algorithm of the Analyst 1.6.2 software (AB Sciex, Darmstadt, Germany) and manually supervised. All other calculations were performed with Excel (Microsoft Office Professional Plus 2010).

### Profiling of Root Exudates

Sample preparation: Exudates were processed according to LC/MS-based metabolite profiling protocol; the residues were reconstituted in 200 µL methanol (supplemented with 0.5 ng ABA-d_6_, 2.5 ng IAA-^13^C_6_, 1 ng JA-d_6_, 0.1 ng JA-Ile-d_2_, 4 ng OPDA-d_5_, 0.4 ng SA, 2.5 ng Z-d_5_, 2.5 ng tZ9R-d_5_, 2.5 ng DHZR-d_5_); incubated for 15 min at room temperature; after centrifugation, the supernatant was processed as described for root extracts, except for the omission of the DEAE-Sephadex SPE.
