# Supplementary material for: Piriformospora indica Stimulates Root Metabolism of Arabidopsis thaliana
Source: Int J Mol Sci. 2016 Jul 8;17(7):1091. doi: 10.3390/ijms17071091 (PMC4964467; doi:10.3390/ijms17071091)
Supplement: Supplementary file 1 [file ijms-17-01091-s001.zip › ijms-133721-Supplementary Materials/ijms-133721-supp-final.pdf]

Supplementary Materials: *Piriformospora indica* Stimulates Root Metabolism of *Arabidopsis thaliana*

Nadine Strehmel, Susann Mönchgesang, Siska Herklotz, Sylvia Krüger, Jörg Ziegler and Dierk Scheel

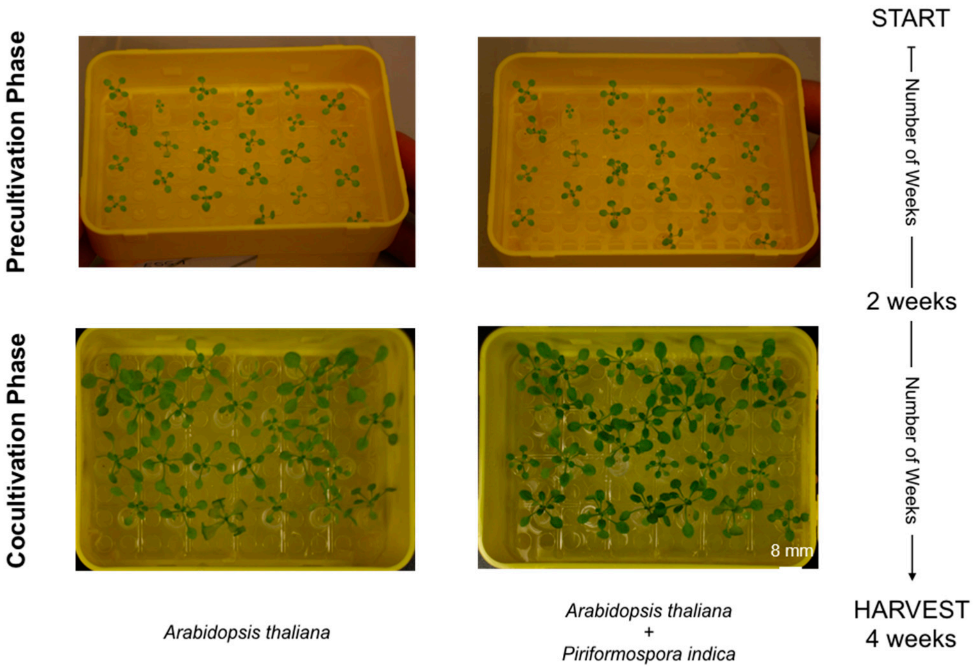

Figure S1. Plant cultivation system for the co-cultivation of *P. indica* with *A. thaliana*. At the beginning of the precultivation stage, surface sterilized *A. thaliana* Col-0 seeds (45 min using 8% sodium hypochlorite and 12.3% hypochloric acid) were sown into 200 µL PCR-tubes filled with agar (0.8% (w/v) Gelrite, 0.5% (w/v) sucrose, 0.5× Murashige-Skoog-Medium (MS) medium and cut at the bottom with a plier so that the roots can penetrate into the hydroponic medium. Then, tubes were arrayed into a PCR-plate and placed into a yellow tip box filled with 180 mL MS medium and 0.5% (w/v) sucrose. Finally, the transparent lids of the boxes were closed, sealed with leukoplast (Duchefa, Haarlem, The Netherlands), and the boxes incubated in a growth cabinet under short day conditions (23/21 °C, photoperiod ~130 $\mu E \cdot m^{-2} s^{-1}$and 8/16-h day/night) for two weeks (pre-cultivation phase). At the beginning of the co-cultivation phase, the cut PCR plate with the plantlets was transferred to a new box containing 180 mL fresh MS medium, 0.5% (w/v) sucrose and a fungal plug (diameter: 8 mm) with *P. indica*, and co-cultivated for additional two weeks under short day conditions (co-cultivation phase).

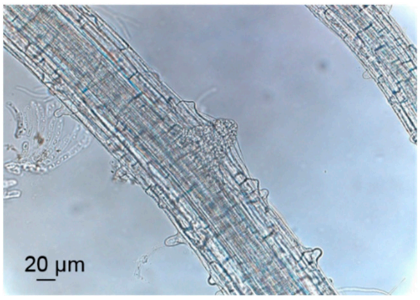

(**A**)

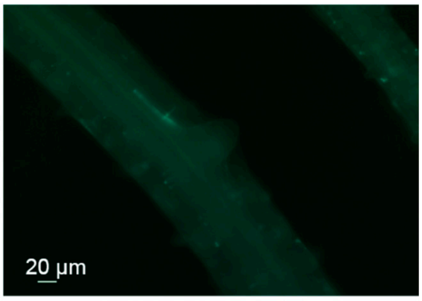

(**B**)

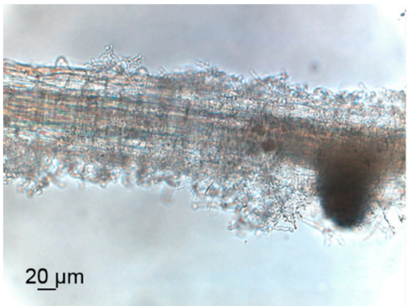

(**C**)

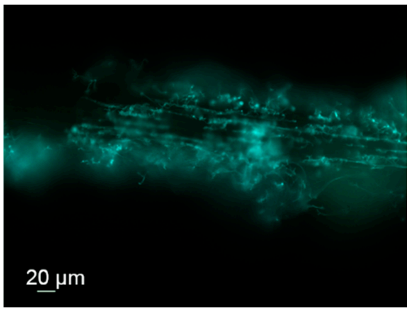

(**D**)

Figure S2. Microscopic images of inoculated and non-inoculated roots of four week-old plants. (**A**) brightfield image of a non-inoculated root; (**B**) fluorescence image of a non-inoculated root; (**C**) brightfield image of an inoculated root with non-GFP labeled *P. indica*; (**D**) fluorescence image of an inoculated root with non-GFP labeled *P. indica.* For the fluorescence microscopy images, two exposure times were used (3 s and 26 s).

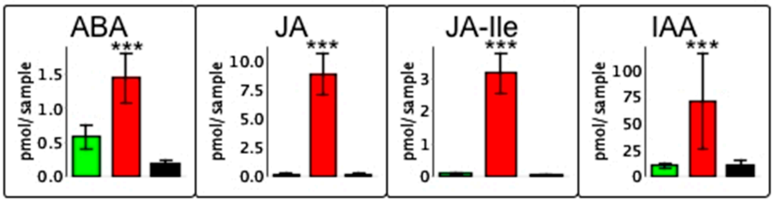

(**A**)

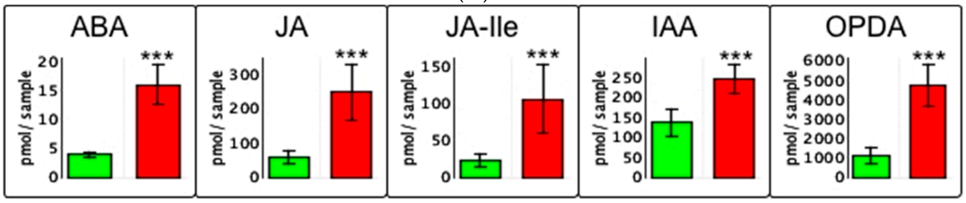

(**B**)

Figure S3. Targeted phytohormone analysis of *A. thaliana* root exudates (**A**) and roots (**B**). Individual sample groups are colour-coded. Green: *A. thaliana*; red: *A. thaliana* + *P. indica*; black: *P. indica*. ABA: abscisic acid; JA: jasmonic acid; JA-Ile: jasmonylisoleucine; IAA: indole-3-acetic acid; OPDA: 12-oxo-phytodienoic acid. Significance analysis of differences between both sample groups (*A. thaliana* and *A. thaliana* + *P. indica*) was performed by *t*-test: ***, $p \leq 0.001$.

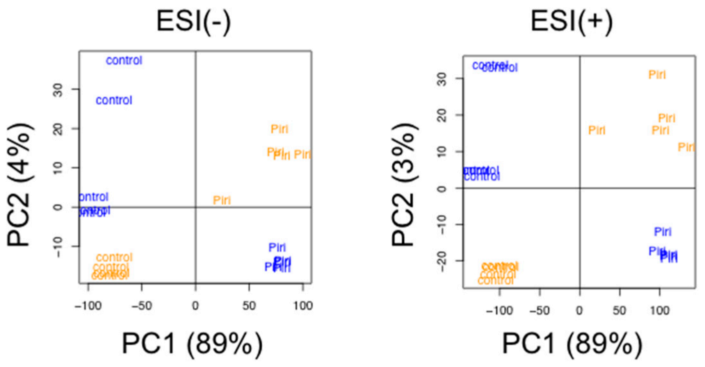

Figure S4. PCA of UPLC/ESI(+/−)-QTOFMS data obtained from root exudates at negative ESI(−) and positive ESI(+) ionization. The two experiments are color-coded. Blue: experiment 1; Orange: experiment 2.

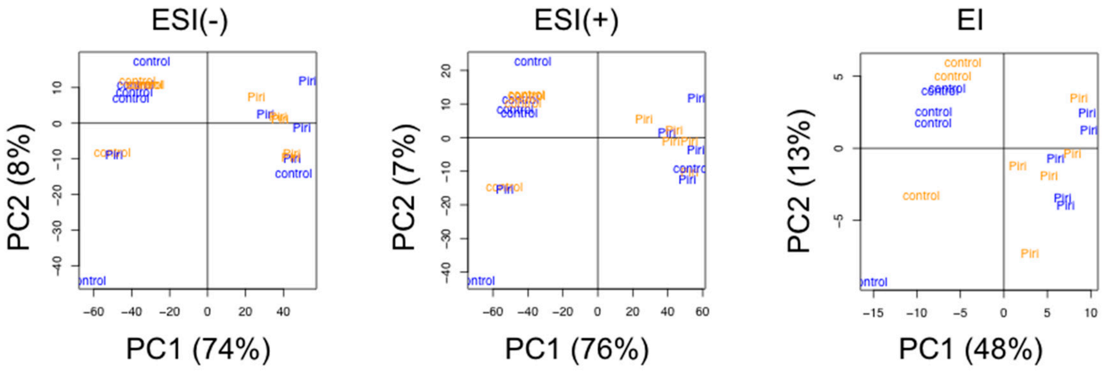

Figure S5. PCA of UPLC/ESI-QTOFMS and GC/EI-QMS data obtained from roots. Negative ionization mode: ESI(−); positive ionization mode: ESI(+); electron impact ionization: EI.

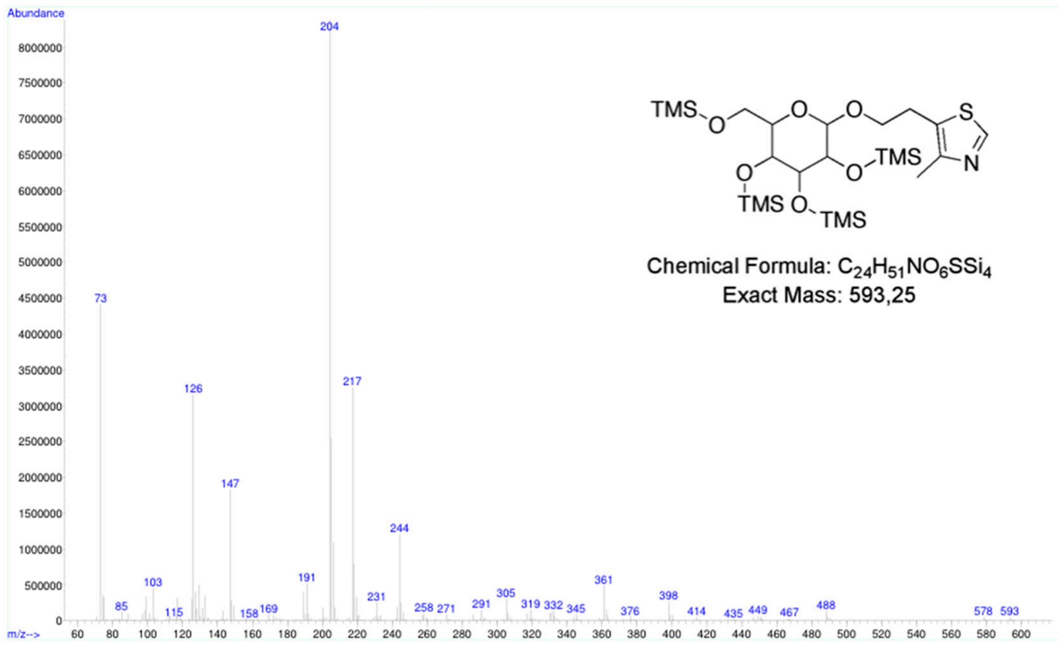

Figure S6. Electron impact mass spectrum of Thiamine hexoside (4TMS) extracted from a pool of non-inoculated roots.

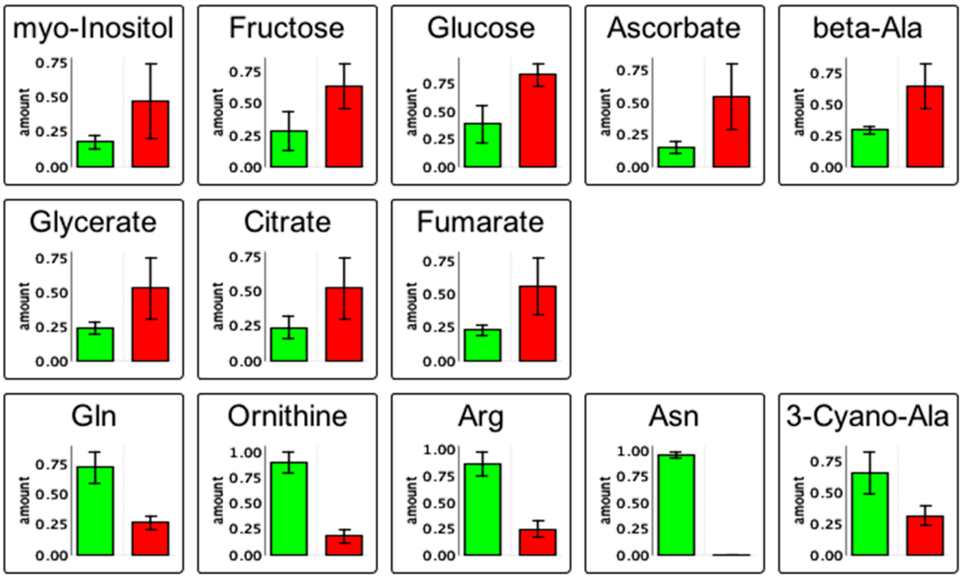

Figure S7. Differentially detected metabolites in leaves of *A. thaliana* with respect to primary metabolism. Candidates were retrieved from a two-sided *t*-Test ($p < 0.01$). Green: A. thaliana; red; *A. thaliana* + *P. indica*.

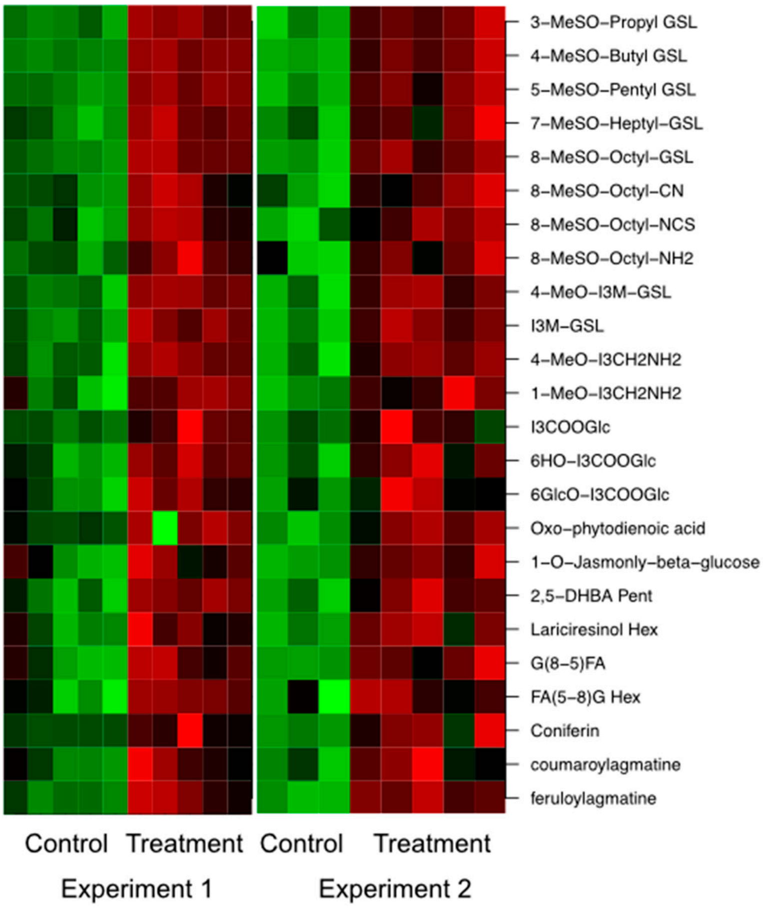

Figure S8. Differentially detected metabolites in leaves of *A. thaliana* with respect to secondary metabolism across to independent biological experiments. Candidates were retrieved from a two-sided *t*-test ($p < 0.01$).

Table S1. Overrepresented GO terms and KEGG pathways among upregulated *A. thaliana* root transcripts 14 dpi.
